# Effect of Biodegradable Nonwoven Mulches from Natural and Renewable Sources on Lettuce Cultivation

**DOI:** 10.3390/polym16071014

**Published:** 2024-04-08

**Authors:** Paula Marasovic, Dragana Kopitar, Tomislava Peremin-Volf, Marcela Andreata-Koren

**Affiliations:** 1 Department of Textile Design and Management, Faculty of Textile Technology, University of Zagreb, Prilaz Baruna Filipovica 28a, 10000 Zagreb, Croatia; paula.marasovic@ttf.unizg.hr; 2 Department for Agriculture, Krizevci University of Applied Sciences, Milislava Demerca 1, 48260 Krizevci, Croatia; tperemin@vguk.hr; 3 Department of Management in Agriculture, Krizevci University of Applied Sciences, Milislava Demerca 1, 48260 Krizevci, Croatia; mkoren@vguk.hr

**Keywords:** hemp, jute, viscose, PLA, nonwoven mulches, lettuce yield, soil quality, weed control efficiency

## Abstract

Numerous research showed that mulching with conventional agro foils elevates soil temperature and promotes plant growth, but negatively influences soil health and brings environmental concerns. Most of the published research on nonwoven mulches for plant cultivation includes nonwoven fabrics produced by extrusion processes providing nonwoven fabric structures similar to films. A limited number of studies investigate the impact of nonwoven mulches produced by a mechanical process on the cards and bonded by needling on plant cultivation. For this study, nonwoven mulches of mass per unit area of 400 g m^−2^ made from jute, hemp, viscose (CV), and polylactide (PLA) fibers were produced on the card bonded by needle punching. The field experiment was conducted two consecutive years in a row, in spring 2022 and 2023, by planting lettuce seedlings. The nonwoven mulches maintain lower temperatures and higher soil moisture levels compared to agro foil and the control field. The fibrous structure and their water absorption properties allow natural ventilation, regulating temperatures and retaining moisture of soil, consequently improving soil quality, lettuce yield, and quality. The fiber type from which the mulches were produced, influenced soil temperature and humidity, soil quality, and lettuce cultivation. The nonwoven mulches were successful in weed control concerning the weediness of the control field. Based on the obtained results, the newly produced mulches are likely to yield better results when used for the cultivation of vegetables with longer growing periods. Newly produced biodegradable nonwoven mulches could be an eco-friendly alternative to traditional agro foil, minimizing environmental harm during decomposition. The obtained results suggest that the newly produced mulches would be even more suitable for growing vegetables with longer growing seasons.

## 1. Introduction

Mulching is an agricultural practice that provides benefits such as reducing soil evaporation, conserving moisture, controlling temperature, and suppressing weeds. Early-season mulching can effectively manage transpiration and soil moisture. This practice not only improves soil properties and nutrient cycling but also serves as an eco-friendly alternative to methods of weed control by using pesticides and insecticides, essential for sustainable farming. Furthermore, mulching could be valuable for weed control, moisture retention, and ultimately plant growth [1,2,3].

For over half a century, traditional low-density polyethylene (LDPE) agro foil mulches have been used to modify the soil microclimate and soil composition directly affecting soil microbial activity and populations and treating ecosystems through microplastic pollution by leaving fragments in the soil during and after the use [4]. Microplastic pollution has become a pressing global concern with environmental, economic, and societal impacts, affecting both land and aquatic ecosystems and posing a threat to biodiversity. Though the implications of microplastics in land environments are not fully comprehended, several studies reveal that plastic mulch film debris significantly alters various soil properties, including moisture content, porosity, pH, organic matter, and water-holding capacity, ultimately affecting soil quality and crop yield. The challenge lies in the non-biodegradability of plastic mulch foils, primarily composed of low-density polyethylene, which necessitates their removal from fields and drives the need for environmentally friendly alternatives [5,6,7].

The use of natural fibers and natural-based polymers, such as jute, polylactic acid (PLA), viscose, hemp and similar has the potential to positively affect soil health, moisture retention, and weed suppression and could increase crop yield with improved crop quality. An Eastern India experiment showed the benefits of jute mulch on crop productivity, nutrient enrichment, and soil microbes [8]. The study conducted by Zawiska and Siwek found that biodegradable PLA film and conventional polypropylene agro foil mulches influenced tomato attributes such as soluble sugar and dry matter concentrations [9]. Studies show that jute mulches improve soil nutrient dynamics, enhancing organic matter, nitrogen, phosphorus, and potassium content over non-mulched plots. In contrast, viscose nonwoven mulches are useful in dry areas where water conservation is important for crop yield because of their sorption qualities, quick biodegradation, and capacity to retain soil moisture [10,11].

Nonwoven mulches from bast fibers, including hemp and flax, offer a compelling alternative to conventional plastic mulches. These mulches excel in weed suppression by obstructing light transmission, impeding weed growth through photosynthesis inhibition, and gradually decomposing upon contact with soil [12]. Moreover, the versatility of nonwoven mulches extends to their ability to modulate temperature, fostering an optimal microclimate for root and plant development, thereby enhancing overall crop productivity [13,14]. Studies have shown that hemp and flax-based mulches provide effective weed control while contributing to soil health through their biodegradable nature and favorable thermal properties.

Plastic mulches, effective in elevating soil temperature and promoting plant growth, entail environmental concerns and are less conducive to soil health. Compared to traditional plastic mulches, nonwoven fabrics present distinct advantages related to their impact on soil temperature and moisture dynamics. For instance, nonwoven jute mulches maintain moisture retention and weed suppression, addressing critical agricultural needs without compromising soil integrity [15,16,17,18,19]. Furthermore, the biodegradability of nonwoven mulches aligns with sustainable agricultural practices, offering a viable solution to mitigate plastic pollution in agroecosystems.

According to the available literature on fiber biodegradation, natural fibers like jute and hemp exhibit faster degradation rates compared to fibers such as viscose and PLA. The viscose mulches offer good sorption properties and relatively fast biodegradation, but PLA mulches degrade more slowly [20]. For instance, nonwoven jute mulches degrade rapidly during 6–8 months of exposure, whereas hemp-based nonwoven mulches can decompose within 1–3 months [20]. Extended research revealed that hemp and jute mulches degraded within a similar timeframe of around 8 to 10 months, while viscose mulches degraded within 6 to 8 months. PLA mulches exhibited a significantly slower degradation [21]. These findings underscore the importance of considering material properties and degradation characteristics when selecting mulching materials for sustainable agricultural practices.

The choice of mulching material plays a pivotal role in shaping soil conditions and microbial communities. While plastic mulches negatively influence microbial biomass and nutrient cycling, nonwoven mulches foster microbial diversity and enzymatic activity, contributing to soil ecosystem health [13,22,23,24,25,26]. Notably, the biodegradation of nonwoven mulches enhanced soil organic carbon content and aggregate stability, underscoring their ecological significance [27,28]. Available research showed that biodegradable plastic mulches exhibit varying effects on soil properties, emphasizing the need for comprehensive research to assess their long-term impact on soil health [29,30]. Previous studies have highlighted the role of nonwoven mulches in enhancing soil quality and promoting sustainable agricultural practices, reaffirming their value as an environmentally friendly alternative to conventional plastic mulches.

Based on the available literature, mulching with nonwoven fabric made of natural fibers should positively affect the plants and the soil, increasing crop yields and soil quality. Most of the published research on nonwoven mulches for plant cultivation includes nonwoven fabrics produced by extrusion processes (spun bond or melt blown process), which provide nonwoven fabrics of small surface masses and thickness, i.e., structures similar to films. Few researches examine the effects of mechanically manufactured nonwoven mulches on cards bonded by needling. In those studies, nonwoven mulches were made mostly of jute fibers. Research on the impact of polylactic acid (PLA) biopolymer mulches on plant growth in all studies was produced by the extrusion processes. In this research, nonwoven mulches from jute, hemp, viscose (CV) and polylactide (PLA) fibers were produced by mechanical process on cards and bonded by needle punching, providing the voluminous fibrous structure. The influence of biodegradable nonwoven mulches on lettuce cultivation was investigated. The investigation includes results of the effect of the nonwoven mulches on the soil temperature and moisture, soil quality and weed control.

## 2. Materials and Methods

**Figure 1:**
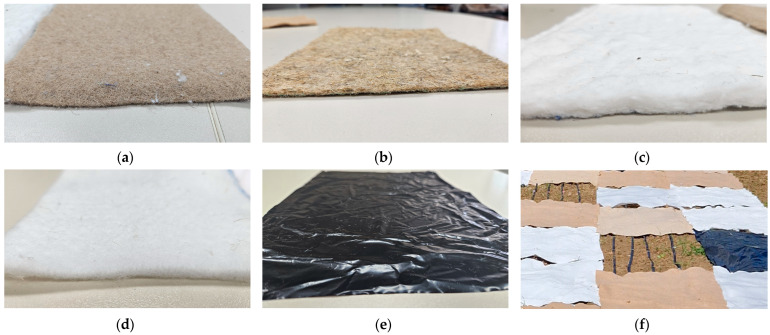
Nonwoven mulches made of (**a**) jute, (**b**) hemp, (**c**) viscose, (**d**) polylactide (PLA) fibers, (**e**) traditional agro foil, and (**f**) control field.

The field test was realized at Donji Laduc, Croatia (45°53′ N, 15°44′ E) where the climate is according to Köppen–Geiger’s humid continental (Dfa) classification. Four samples of nonwoven mulches from jute, hemp, viscose (CV) (Derotex, Wielsbeke, Belgium)and polylactide (PLA) fibers (NatureWorks BV, Plymouth, MN, USA) in a nominal mass per unit area of 400 g m^−2^ were produced (Figure 1). A commercially available black PE agro foil (28.17 g m^−2^) and control field (bare soil abbreviated in text as CF) were included in the experiment to compare outcomes with traditionally used mulching materials and bare soil.

The nonwoven mulches were produced using a low-cost nonwoven fabric production process. The webs of nonwoven fabric are produced by a mechanical process on a card and laid down on cross lappers where the number of layers depends on nonwoven fabric mass per unit area (desired mass was 400 g m^−2^). The layered webs were bonded through the needle-punching process. The physical–mechanical properties of nonwoven mulches and agro foil are given in Table 1.

**Figure 2:**
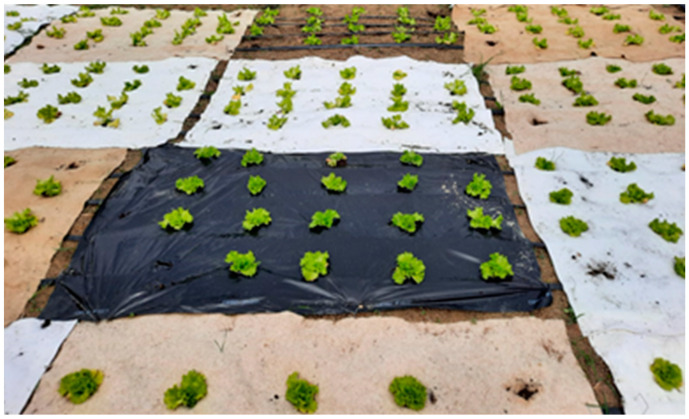
The nonwoven mulches, agro foil, and control field placed on the soil in the four replication plots.

**Figure 3:**
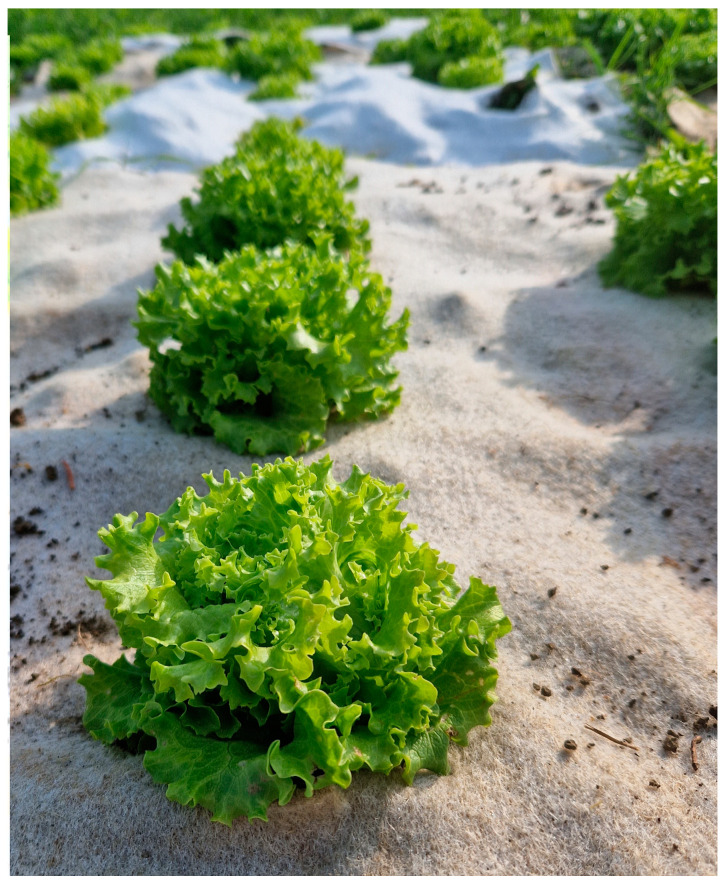
Gentilina lettuce (*Lactuca sativa* L.).

The nonwoven mulches and agro foil of 2.25 m^2^ (1.5 × 1.5 m) were placed on the soil according to randomized block design with four replications where an irrigation system, drip by drip, was also installed (Figure 2). At the end of April 2022 and the beginning of May 2023, twenty seedlings of Gentilina lettuce (*Lactuca sativa* L.) on each plot were planted (Figure 3). The experiment was conducted two consecutive years in a row.

Gentilina lettuce is chosen for planting due to bolting resistance in regions or seasons where air temperatures are warmer or days are longer, which describes the climate of the region where the experiment was conducted (Figure 3). The recommended time for seedling is from March to October, especially from March to July for outdoors. The recommended time from seedling to harvesting is 28 days and for full-size lettuce 45 days [31].

During 50 days of trial, valid for both years, soil temperature and moisture were recorded once per week. A PMS-714 (Lutron Electronic Enterprise Co., Taiwan) soil moisture meter was used to measure the soil moisture at a depth of 15 cm. The soil temperature was measured with a bi-metal dial thermometer from the Fisher brand at the same depth.

The hydro-meteorological station closest to the area’s mulch exposure site provided air temperature and humidity during the experiment. During the experiment, lettuce growth and development were recorded by measuring lettuce head diameter and height with an angle square ruler.

At the end of the experiment (after 50 days), soil samples were collected beneath each mulch type and the control field from four replication plots. Soil samples were taken from each experimental field (hemp, jute, viscose, PLA, agro foil, and the control field) using a cylindrical agrochemical probe for soil sampling at 0–30 cm depth.

The soil’s physicochemical properties and microorganism colonies beneath nonwoven mulches and the control field were measured according to standard protocols. The soil’s pH value was tested according to the ISO 10390:2021 standard [32]. The organic carbon content was calculated based on the total humus value. Using the bichromate method, humus is determined where soil organic matter was wet-burned using K-bichromate.

Total nitrogen content was determined using the Kjeldahl method. To determine the easily accessible (plant-available) P_2_O_5_ and K_2_O, the AL method was employed. This method involved extracting phosphorus and potassium from the soil using an ammonium lactate solution with a pH of 3.75. Easily accessible potassium refers to its water-soluble and exchangeable sorbed form, while easily accessible phosphorus represented the various forms of phosphorus in the soil that could pass into weak acid, base, or salt solutions.

According to the ISO 10694:1995 standard [33], the total organic carbon (TOC) was measured and presented as a percentage of dry matter (105 °C-dried to constant mass).

The total population of bacteria and fungi in the soil beneath the mulches was tested using traditional microbiological techniques. All soil samples were mixed in a sterile physiological solution for five minutes. Nutrient agar (NA) was used to assess the total bacterial count, while Czapek agar was utilized to determine the total fungal count. Each sample was analyzed three times. Once the cultures had developed on the nutrient media, the colonies were counted, and the CFU (colony-forming unit) values per gram of soil were determined for each group of microorganisms.

To evaluate the ability of the mulches to control weeds (weediness), on the day of harvesting, after the lettuce was collected, the weeds above nonwoven mulches, PE agro foil, and on the control field were cut. The collected weeds were dried to an absolute dry and weighed on an analytical balance. The percentage of weeding of nonwoven mulches and agro foil was compared with the control field, where the share of weeds in the control field was 100%.

An analysis of the lettuce to evaluate the quality through a quantity of moisture and dry matter, nitrogen, phosphorus, potassium, calcium, magnesium and iron was obtained. The moisture content and dry matter were obtained by drying at 105 °C. Nitrogen content was tested by the Kjeldahl method, phosphorus by spectrophotometry, and potassium by flame photometry. Ca, Mg and Fe were read on AAS—PinAcle 900F (PerkinElmer, Traiskirchen, Austria). All elements are presented as a percentage of dry matter, except for iron which is presented as mg/kg of dry matter.

## 3. Results and Discussion

### 3.1. The Effect of Mulch on Soil Temperature and Moisture

The average soil temperature and moisture for years 2022 and 2023, with the associated average air temperature and air humidity, are presented in Table 2 and Table 3. The soil temperatures beneath nonwoven mulches are mostly lower than beneath the conventional agro foil and control field, i.e., nonwoven mulches affect the soil temperatures but depend on air temperatures (Table 2).

In the first year of the study, the soil temperature beneath nonwoven mulches was lower than on the control field for the first four weeks. Over the subsequent two weeks, the temperatures were similar to those on the control field, and by the final week, they surpassed the control field’s temperature. In the second year of the experiment, temperatures beneath nonwoven mulches closely aligned with those on the control field.

On average, soil temperatures under nonwoven mulches were lower compared to agro foil, showing a difference ranging from 0.5 °C to 2.5 °C in May 2022 and from 0.8 °C to 1.6 °C in June 2022. However, the temperature differences between nonwoven mulches and agro foil in the second year were markedly reduced, ranging from 0.2 °C to 0.8 °C in May 2023 and from 0.7 °C to 1.0 °C in June 2023. The most significant temperature disparities between agro foil and nonwoven mulches were observed for viscose mulches, 12.0% in 2022 and 5.4% in 2023.

Generally, nonwoven mulches tended to maintain lower temperatures compared to agro foil. Compared to bare soil, the temperatures under nonwoven mulches were more similar to agro foil, influenced by air temperature and rainfall. May 2023 experienced cooler temperatures with a rainfall of 157.8 mm, while May 2022 had higher temperatures with rainfall measuring only 53.9 mm.

Throughout the two-year experiment, soil moisture remained relatively consistent due to the regulated irrigation system (Table 3). Soil moisture levels were consistently higher under nonwoven mulches than agro foil and the control field. The nonwoven mulches’ fibrous structure and absorption properties of fibers absorbed water until balance with soil/air moisture was reached, and then the structure maintained higher soil moisture than under an impermeable agro foil. Specifically, in May 2022, the difference in soil moisture between nonwoven mulches and agro foil ranged from 0.6% to 2.4%, and in June 2022, it ranged from 0.1% to 0.9%. In the second year, the soil moisture variance between nonwoven mulches and agro foil was between 0.6% and 1.2% in May 2023, and between 1.1% and 2.5% in June 2023.

Interestingly, during the first week of 2022 and 2023, soil moisture under nonwoven mulches was lower than in the control field. This could be attributed to the absorption characteristics of nonwoven mulches, which absorb more water from the irrigation system than the soil during the lettuce-planting phase. However, after this initial week, the soil moisture under the nonwoven mulches equilibrated with field conditions and remained higher than the control field for the duration of the experiment.

The exception is the first week of June 2023, where probably previously mentioned rainfalls influenced the soil moisture on the control field. For a more detailed conclusion, it is necessary to investigate the absorption characteristics of nonwoven mulches produced by different fiber types at certain air and soil temperatures and humidity.

A two-way ANOVA was conducted to assess the statistical significance of temperature and soil moisture under various mulches and in the control field during 2022 and 2023. The ANOVA revealed significant differences between temperatures and soil moisture, and therefore Duncan’s new multiple range test (MRT) was performed (Table 4). In 2022, the statistical differences in soil temperatures between viscose nonwoven mulch soil temperatures regarding PLA, agro foil and the control field were obtained. In the same year, a statistical difference in soil moisture was not found. Contrary to 2022, in the year 2023, no significant differences in soil temperatures and moisture were found. It is a logical result considering irrigation system installation, where the soil moisture was controlled and equal conditions beneath nonwoven mulches, agro foil and on the control field were maintained. The obtained results indicate the impact of precipitation, which, as already stated, was significantly greater in 2023. For a more detailed conclusion, it is necessary to investigate the influence of the amount of water absorbed by nonwoven mulches and their ability to maintain soil moisture and heat, i.e., soil-nonwoven mulch thermodynamics.

**Figure 4:**
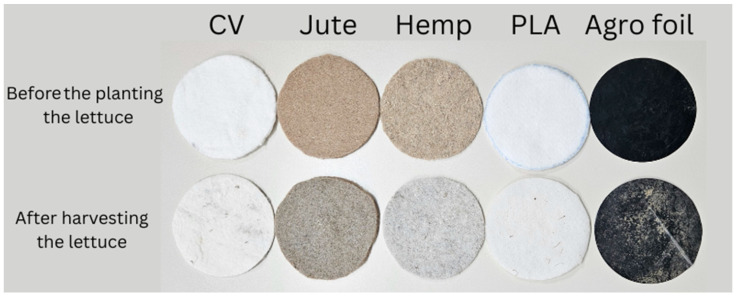
Nonwoven mulches and agro foil before lettuce planting and after lettuce harvesting.

Due to the effect of environmental conditions (weather, sun, rainfalls, soil) on mulches by visual examination of mulches before planting and after harvesting of the lettuce, changes in the appearance were observed (Figure 4). The noticeable change was the color difference for jute and hemp nonwovens compared to other mulches. Soil deposition onto the nonwoven surfaces during the experiment significantly altered their appearance. As the lettuce crops matured, the soil, rich in organic matter and minerals, adhered to the surfaces of the nonwoven materials, imparting a distinct earthy patina. Secondly, the UVA radiation emitted by the sun, a ubiquitous presence in outdoor agricultural environments, exerted its influence on nonwoven materials. Exposure to ultraviolet rays inevitably led to photodegradation, causing a shift in coloration and surface texture. The jute and hemp nonwovens, being natural fibers, were particularly susceptible to this phenomenon. Moreover, the exposure to moisture (rain, dew and other rainfall) affected the structure and overall appearance of nonwovens.

### 3.2. The Effect of Mulch on the Lettuce Growth and Yield

**Figure 5:**
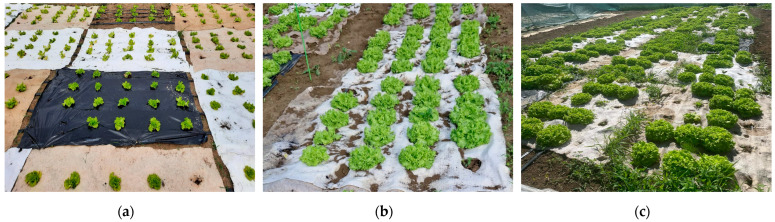
Phases of lettuce growth: (**a**) after planting, (**b**) after one month, (**c**) on the day of harvest (50 days).

To determine the impact of the nonwoven mulches on lettuce growth dynamics, the height and diameter of the lettuce were regularly measured from the time of planting until harvest (Figure 5). Measurements were taken every ten days throughout both years of the experiment, with the collected data presented in Table 5 and Table 6.

Upon harvesting in 2022, lettuce plots covered with agro foil exhibited the largest average rosette diameter, while those covered with mulch made of PLA fibers showed the smallest diameter. The results for the subsequent year were different: plots covered with PLA mulches had the largest rosette diameter, whereas those covered with jute mulches had the smallest. ANOVA statistical analyses indicated no significant differences in average rosette diameter in 2022 (*p* = 0.12) and 2023 (*p* = 0.23).

In 2023, the rosette height of lettuce exceeded that of 2022 for all mulches (Table 6). At the time of harvest in 2022, the tallest rosette heights of lettuce were found in plots covered with jute, while the shortest heights were observed in plots with nonwoven mulch made from PLA fibers. Conversely, in 2023 the tallest rosette height was recorded in lettuce plots mulched with hemp nonwoven fabrics, whereas the shortest height was measured in the control field. In both 2022 and 2023, ANOVA results indicated no significant differences in average rosette height (*p* = 0.56; *p* = 0.10) at the significance level of *p* = 0.05. Based on the results, it can be concluded that differences in lettuce heights and diameters were not significant, and that the impact of mulches on lettuce growth dynamics was not observed.

The mass of lettuce, grown on soil covered with mulches, was generally higher in both 2022 and 2023 compared to the control field (Table 7). However, in 2022, the mass of lettuce covered with PLA nonwoven mulch was slightly lower than the control.

In 2022, lettuce grown in fields covered with viscose and jute mulches exhibited greater mass, while those in fields with hemp mulches were comparable to those covered with agro foil. In contrast, in 2023, the mass of lettuce grown on the field covered with viscose and hemp mulches surpassed the mass of lettuce grown on the field covered with agro foil. Based on the results, the influence of nonwoven mulches on lettuce mass during the two years of the experiment was not determined. Both in 2022 and 2023, the ANOVA results did not show significant differences in the mass of rosettes between mulched and untreated plots (*p* = 0.99; *p* = 0.10) at a significance level of *p* = 0.05.

**Figure 6:**
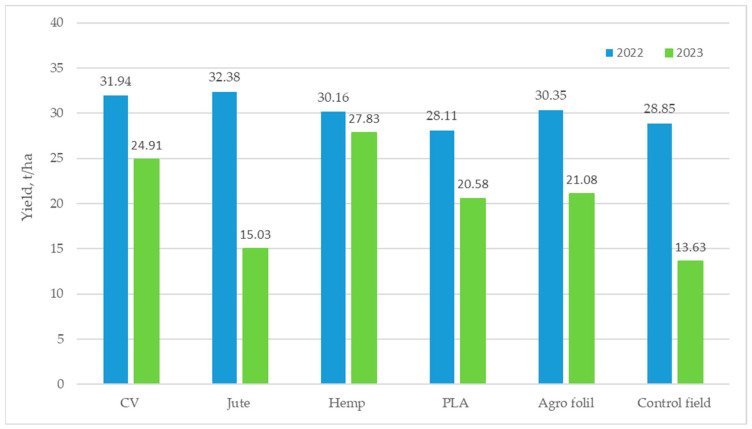
Lettuce yields for years 2022 and 2023.

The yield of lettuce was determined based on the mass of fresh lettuce (expressed in tons) and the plant population per unit area (expressed in hectares). Generally, a higher lettuce yield was recorded in 2022 regarding to 2023 (Figure 6).

Although in the first year of the experiment the lettuce grown on the plots covered with agro foil had the largest diameter and height, the plots mulched with jute (6.3%) and viscose (5.0%) nonwoven mulches gave higher yields compared to agricultural film. The yield from plots covered with agro foil was only 0.6% higher than those covered with hemp mulches. The lowest lettuce yield was observed in plots covered with PLA mulches, which was even lower than that of the control field. In 2023, the highest lettuce yield was achieved in plots covered with hemp mulch, registering at 27.83 t/ha, while the lowest yield was recorded in the control field at 13.63 t/ha.

In 2022, lettuce grown on agro foil initially showed greater height but had lower values just before harvest, compared to viscose, jute nonwoven mulches, and the control field. As the experiment progressed, rising air temperatures under the foil decreased soil moisture, making conditions less favorable than nonwoven mulches. Nonwoven mulches maintained lower soil temperatures and slightly higher moisture levels, offering optimal lettuce growth conditions over agro foil. Similarly, in the second year of the experiment, nonwoven mulches appeared more beneficial for lettuce development. Unlike agro foil, nonwoven mulches are composed of open and closed micropores, creating natural ventilation. i.e., better regulating temperatures and retaining moisture, which improves soil quality, and consequently, lettuce yield and quality. Using nonwoven mulches, lettuce yield surpassed the yield of the control field, highlighting mulch’s significant role in lettuce development.

When lettuce yield grown on nonwoven mulches is compared to agro foil, it could be concluded that lettuce yield is influenced by varying weather conditions across the experiment years. In 2022, lower yields were obtained with hemp (0.6%) and PLA (7.9%) mulches, while in 2023, jute (40.3%) and PLA (2.4%) mulches showed lower yields. Notably, hemp mulch consistently improved lettuce growth and yield across the climatically diverse years.

### 3.3. The Effect of Mulch on the Lettuce Nutrient Content

The nutrient content in lettuce grown in the years 2022 and 2023 is presented in Table 8. Mulching practices can influence lettuce nutrient content by affecting soil conditions. Proper mulching practices can help optimize nutrient content and uptake for improved lettuce growth and quality.

In the first year of the experiment, lettuce grown under agro foil and in the control field had the highest nitrogen content at 3.53% of total dry matter (Table 8). In the second year, lettuce under nonwoven mulches had higher nitrogen content compared to the control field. In the first year of the experiment, the content of phosphorus (P), potassium (K), magnesium (Mg), and iron (Fe) was higher under nonwoven mulches compared to conventional agro foil. However, the calcium (Ca) content was lower under nonwoven mulches than on the control field and below agro foil, ranging from 0.04% to 0.21%. In the second year of the experiment, a similar trend was observed. The P content was lower under PLA mulches, and the content of K, Ca, Mg, and Fe was lower under hemp nonwoven mulches compared to other mulches. Due to interactions between nutrients, the availability and uptake of P, K, Mg, and Fe can be interrelated. Imbalances in one nutrient can affect the uptake and utilization of other nutrients.

Considering that most factors that influence nutrition content in lettuce were equal to all planted lettuce, and the only factors that differed were mulch type and environmental conditions, the influence of these parameters is visible. The environmental conditions between the two years of the experiment differed in regard to influencing different soil conditions (soil moisture and temperature, soil microbial activity) due to placing different nonwoven mulches on soil leading to different nutrition content in lettuce.

A mostly positive impact of mulching with nonwoven mulches compared to conventional agro foil is visible. Comparing the nutrition content of lettuce grown on the control field and nonwoven mulches showed that mulches provided higher percentage values of some lettuce nutrients, while in some cases lettuce contents were lower.

### 3.4. The Effect of Mulch on the Soil Quality

The soil nutrient values are mostly higher in soil covered by mulches regarding soil nutrients before the experiment, i.e., lettuce planting, except for potassium oxide (Table 9). Mulches reduce water runoff and soil erosion, which helps to retain soil nutrients in the layer of soil that was taken for testing.

Soil pH was consistent across all plots, including the control field and agro foil. In the first year, the lowest humus and organic carbon percentages were under jute mulches, even lower than in soil covered with agro foil. In the second year, all nonwoven mulches had lower humus and higher organic carbon content compared to agro foil. Total nitrogen was lower under jute and hemp mulches in the first year and remained lower in the second year compared to soil below agro foil.

The results of the soil content suggest that the type of fibers from which the mulches are made could influence the content of nutrients in the soil, the level of humus and carbon. Generally, mulches produced from natural and renewable sources may contribute soil nutrients as they decompose, potentially increasing soil nutrient levels. When mulch decomposes soil, microbial activity increases since the remains are food sources for microbes. Increased microbial activity improves nutrient availability and cycling in the soil. It is assumed that a 50-day period of mulching with nonwoven mulches was too short for the influence of mulches on soil nutrition. The influence of field conditions on soil nutrition is visible since different trends were found regarding the year of lettuce planting. The observed differences in nutrient content indicate that the choice of mulch could play a role in influencing soil fertility and nutrient availability, which can subsequently impact plant growth and overall agricultural productivity.

### 3.5. The Effect of Mulch on the Total Organic Carbon and Number of Microorganisms (Bacteria and Fungi) in Soil

In 2022, the TOC values were generally higher than in 2023 (Table 10). In 2022, the lower TOC regarding agro foil were recorded in the soil covered by hemp (3.29%) and PLA (3.40%) mulches, which were even lower than the control field (3.42%). The TOC values in the soil covered by viscose (3.84%) and jute (3.61%) mulches were higher than in soil covered by agro foil and on control fields. In 2023, contrary to the previous year, only soil covered by jute mulches had higher TOC values regarding agro foil.

The variation in TOC values between the two years suggests that factors beyond mulch type contribute to changes in TOC content. The mulches had different effects in different years, highlighting the complex interactions between mulch type, soil, and environmental conditions. The variation in TOC might be related to the decomposition rates of tested mulches. Some mulches might decompose more slowly, leading to a longer-term impact on TOC values. Obtained data show that the effects of mulch on TOC levels are not consistent and can vary based on multiple factors.

Exposure of natural mulches to field conditions causes their degradation, and the remains are a potential source of food for bacteria and fungi in the soil. Higher food content in soil provides an increase in bacteria and fungi colonies. In both years of the experiment, a significantly higher number of fungi were found in soil below nonwoven mulches. In 2023, the bacterial count in the soil below nonwoven mulches was significantly higher than in 2022.

Nonwoven mulches generally supported higher fungal colonies compared to agro foil, suggesting that they create favorable conditions for microorganism growth by acting as a barrier between soil and the environment. Besides the environment, the degradation process of bast fibers starts with fungi attacking the protective layer; therefore, it is assumed that the decomposition process of jute and hemp mulches has started. The second stage of bast fiber degradation would be bacterial penetration into the fiber and degradation of the fiber cellulose. In 2022, degradation of the bast fibers in nonwoven mulches was slower than in 2023. In 2023, greater precipitation and higher temperatures a few weeks before ending the experiment accelerated the degradation process of bast fiber from nonwoven mulches, i.e., fungi degrade the outer layer of bast fiber and bacteria started to eat cellulose, consequently number of bacterial colonies in soil increases. The viscose and PLA fibers from mulches are more preferred food sources than agro foil, so colonies of fungi and bacteria are greater than below agro foil.

The higher numbers of bacteria and fungi associated with certain nonwoven mulches could be linked to increased organic matter content and nutrient availability provided by mulch degradation. The presence of organic materials in nonwoven mulches like hemp, jute, and viscose might have contributed to the increase in TOC levels. As these mulches break down over time, they release organic matter into the soil, which serves as a nutrient source for microorganisms, contributing to soil organic carbon content. The increase in TOC levels due to the decomposition of nonwoven mulches likely provided additional nutrients for microbial communities.

### 3.6. The Effect of Mulch on the Weed Control

The nonwoven mulches were successful in weed control concerning the weediness of the control field (Table 11).

The best weed suppression was provided by viscose nonwoven mulch and the lowest by hemp mulches. Mulches prevented the development of weeds by creating a barrier between the soil and the environment, preventing the weed’s photosynthesis and yet providing favorable conditions for the development of lettuce. Due to more abundant rainfall and moderate temperatures, the percentage of weeds in 2023 is higher compared to the previous year.

## 4. Conclusions

Newly produced biodegradable nonwoven mulches made from viscose, jute, hemp, and PLA fibers on the card and bonding the fabric by needle punching offer a potential alternative to conventional PE agro foil used in agriculture. During lettuce growth, nonwoven mulches consistently impact soil temperatures and moisture levels. Compared to agro foil, nonwoven mulches lower soil temperatures, whereas mulches made of viscose fibers show the most significant temperature difference. Over the growing lettuce period, from May to June, the difference in soil temperatures between agro foil and nonwoven mulches decreased. Additionally, nonwoven mulches consistently maintain higher soil moisture levels compared to agro foil and control fields.

In 2022, increased lettuce yields were achieved using jute (0.6%) and viscose (7.9%) mulches compared to agro foil. Additionally, jute (0.6%), hemp (5%), and viscose (7.9%) mulches outperformed the control field in terms of lettuce yield. However, lettuce yields differed in 2023. Hemp (40.3%) and viscose (2.4%) mulches yielded more lettuce than agro foil, where all plots mulched by nonwoven mulches yielded more lettuce than on the control field.

Comparing the nutrient content of lettuce grown on the control field and mulched by agro foil to lettuce mulched with nonwoven mulches revealed varying results. Some nonwoven mulches boosted certain lettuce nutrients (phosphorus, potassium, magnesium and iron), while others showed lower levels (nitrogen and calcium) compared to the control field and agro foil.

Results indicate that the choice of degradable mulch could influence soil fertility and nutrient availability, considering the time frame of mulch degradation, which can subsequently impact plant growth and overall agricultural productivity.

In terms of weed control, viscose nonwoven mulch proved the most effective, while hemp mulches were the least effective.

Overall, the study underscores the potential of biodegradable nonwoven mulches as environmentally friendly alternatives to traditional agro foil. These mulches can preserve moisture, reduce soil temperatures, and offer effective weed control. Future research should delve into the effects of biodegradable nonwoven mulches on non-irrigated cultivation, considering their demonstrated benefits in moisture retention and temperature reduction. Also, the influence of nonwoven mulches on cultures with longer growing seasons should be explored considering the time needed for mulch degradation.

## Figures and Tables

**Table 1:** Physical–mechanical properties of nonwoven mulches and agro foil.

Mulch	Parameter	Value
CV	Mass per unit area, g/m^2^	410.8
	Thickness, mm	6.2356
	Air permeability, cm^3^/cm^2^/s	31.5
	Tensile strength in MD, kN/m	0.0933
	Tensile strength in CD, kN/m	0.1312
Jute	Mass per unit area, g/m^2^	468.8
	Thickness, mm	5.1865
	Air permeability, cm^3^/cm^2^/s	126.5
	Tensile strength in MD, kN/m	0.1495
	Tensile strength in CD, kN/m	0.2125
Hemp	Mass per unit area, g/m^2^	473.4
	Thickness, mm	4.1064
	Air permeability, cm^3^/cm^2^/s	199.4
	Tensile strength in MD, kN/m	0.3955
	Tensile strength in CD, kN/m	0.4810
PLA	Mass per unit area, g/m^2^	363.9
	Thickness, mm	5.3825
	Air permeability, cm^3^/cm^2^/s	113.3
	Tensile strength in MD, kN/m	0.4158
	Tensile strength in CD, kN/m	0.4844
Agro foil	Mass per unit area, g/m^2^	28.2
	Thickness, mm	0.0670
	Tensile strength in MD, kN/m	0.4011
	Tensile strength in CD, kN/m	0.6667

Where MD is tensile strength in the machine direction, a CD is a tensile strength in the cross-machine direction, CV is mulch made of viscose fibers, PLA is mulch made of polylactide fibers.

**Table 2:** Soil temperature (°C) beneath nonwoven mulches and on the control field in years 2022 and 2023.

Year	2022							2023						
Month	May				June			May		June				
Date	5	12	16	26	2	6	13	19	25	1	7	16	21	26
CV	13.8	15.3	16.9	18.6	17.1	20.7	18.9	13.7	18.2	17.2	18.2	18.4	20.6	20.2
Jute	14.9	16.8	18.2	19.7	18.1	21.2	19.5	14.1	18.7	17.7	18.7	18.7	21.1	20.4
Hemp	14.8	16.5	18.4	19.5	18.1	21.4	19.7	14.0	18.6	17.5	18.6	18.5	20.9	20.2
PLA	15.3	17.3	18.6	19.8	18.2	21.2	19.6	14.0	18.6	17.6	18.5	18.6	21.0	20.5
Agro foil	16.1	17.9	19.4	21.1	19.1	22.0	20.3	14.3	19.2	18.0	18.9	20.2	21.6	21.1
CF	16.3	17.8	18.6	20.0	18.1	21.2	18.8	13.9	19.2	17.9	18.8	18.5	21.0	19.9
AT, (°C)	15.8	23.0	21.0	19.6	20.2	21.6	22.1	12.9	19.0	20.4	20.6	18.4	27.0	20.6
RH, %	90.0	95.0	86.0	98.0	92.0	87.0	88.0	97.0	94.0	93.0	84.0	83.0	74.0	95.0

Where CF is the control field, CV is mulch made of viscose fibers, PLA is mulch made of polylactide fibers, AT is the average air temperature in °C; RH is relative air humidity %.

**Table 3:** Soil moisture (%) beneath nonwoven mulches and on the control field in years 2022 and 2023.

Year	2022							2023						
Month	May				June			May		June				
Date	5	12	16	26	2	6	13	19	25	1	7	16	21	26
CV	31.2	39.4	45.5	46.1	43.2	46.4	46.1	24.3	27.4	39.7	44.1	23.0	37.4	43.3
Jute	29.4	42.1	43.2	45.8	45.8	46.7	45.1	23.6	26.8	36.7	46.4	22.9	41.7	42.9
Hemp	31.0	43.3	47.8	45.8	46.5	44.9	43.8	25.0	27.2	38.8	44.3	23.8	40.3	44.5
PLA	34.2	39.2	45.1	45.2	45.9	45.3	43.3	23.9	24.4	35.2	42.3	22.8	41.9	45.7
Agro foil	26.6	40.0	45.8	45.8	44.4	46.4	43.9	23.5	25.7	33.2	43.6	23.1	33.7	45.4
CF	42.9	37.1	43.0	43.0	43.1	47.3	45.5	24.7	25.8	48.9	42.0	22.6	27.7	43.5
AT, (°C)	15.8	23.0	21.0	19.6	20.2	21.6	22.1	12.9	19.0	20.4	20.6	18.4	27.0	20.6
RH, %	90.0	95.0	86.0	98.0	92.0	87.0	88.0	97.0	94.0	93.0	84.0	83.0	74.0	95.0

Where CF is the control field, CV is mulch made of viscose fibers, PLA is mulch made of polylactide fibers AT is the average air temperature in °C; RH is relative air humidity %.

**Table 4:** Duncan statistical analysis of average soil temperature and moisture beneath the mulches and on the control field during experiment in 2022 and 2023.

Samples	2022	2023
	Soil temperature	
CV	17.33b	18.08a
Jute	18.33ab	18.33a
Hemp	18.35ab	18.43a
PLA	18.57a	18.48a
Agro foil	19.41a	19.06a
CF	18.67a	18.48a
	Soil moisture	
CV	45.50a	34.17a
Jute	42.53a	34.43a
Hemp	43.31a	34.86a
PLA	42.44a	33.75a
Agro foil	41.84a	32.62a
CF	43.16a	33.60a

Where CF is the control field, CV is mulch made of viscose fibers, PLA is mulch made of polylactide fibers. Different letters indicate significant differences according to Duncan test (*p* ≤ 0.05).

**Table 5:** Lettuce average diameter (cm) for the years 2022 and 2023.

Year	2022					2023			
Month	May			June		May		June	
Date	5	16	26	6	13	19	7	16	26
CV	10.62	10.33	17.32	21.38	21.86	10.73	19.67	24.17	26.71
Jute	10.80	10.83	17.58	20.82	21.11	10.98	16.68	23.06	24.04
Hemp	10.16	11.96	18.09	20.31	20.88	11.65	17.66	25.46	26.20
PLA	9.70	10.70	16.13	18.59	18.88	12.08	17.90	25.54	27.07
Agro foil	9.60	12.64	18.53	22.31	23.21	9.71	18.59	25.11	25.95
CF	10.24	12.62	19.14	22.21	21.66	11.64	18.13	22.33	23.69

Where CF is the control field, CV is mulch made of viscose fibers, PLA is mulch made of polylactid fibers.

**Table 6:** Lettuce height (cm) for years 2022 and 2023.

Year	2022					2023			
Month	May			June		May		June	
Date	5	16	26	6	13	19	7	16	26
CV	8.71	7.36	12.35	15.30	14.95	5.89	10.34	14.39	14.40
Jute	7.93	8.58	13.01	15.53	16.34	5.81	9.55	13.20	13.01
Hemp	7.79	8.94	13.90	14.51	14.53	5.63	9.39	13.90	14.91
PLA	8.04	8.98	13.00	15.43	14.14	6.01	10.80	14.96	14.73
Agro foil	7.74	9.05	13.96	16.60	14.83	5.54	10.90	15.45	14.34
CF	8.18	8.79	12.83	15.80	16.04	6.11	9.86	12.56	12.95

Where CF is the control field, CV is mulch made of viscose fibers, PLA is mulch made of polylactide fibers.

**Table 7:** Average mass of lettuce at the time of harvest in the years 2022 and 2023.

		Mass of Rosettes, g	
Year	Statistical Parameters	2022	2023
CV	Mean value, g	287.8	224.4
	Standard deviation, g	70.0	65.6
	Coefficient of variation, %	24.3	29.3
Jute	Mean value, g	291.7	135.4
	Standard deviation, g	149.5	65.6
	Coefficient of variation, %	51.3	48.5
Hemp	Mean value, g	271.7	250.8
	Standard deviation, g	62.2	71.9
	Coefficient of variation, %	22.9	28.7
PLA	Mean value, g	253.3	185.4
	Standard deviation, g	118.5	66.5
	Coefficient of variation, %	46.8	35.9
Agro foil	Mean value, g	273.4	189.9
	Standard deviation, g	50.9	78.4
	Coefficient of variation, %	18.6	41.3
CF	Mean value, g	259.9	122.8
	Standard deviation, g	113.4	22.1
	Coefficient of variation, %	43.6	18.0

Where CF is the control field, CV is mulch made of viscose fibers, PLA is mulch made of polylactide fibers.

**Table 8:** Nutrient content in lettuce for years 2022 and 2023.

	Moisture, %	Dry Matter, %	N, % (d.m.)	P, % (d.m.)	K, % (d.m.)	Ca, % (d.m.)	Mg, % (d.m.)	Fe, mg/kg (d.m.)
Year	2022							
CV	95.82	4.18	3.36	0.32	5.45	1.77	0.25	94.50
Jute	95.56	4.44	2.86	0.31	5.49	1.71	0.29	144.95
Hemp	95.79	4.21	3.21	0.31	5.68	1.60	0.31	94.55
PLA	95.94	4.06	2.85	0.34	5.36	1.72	0.31	134.75
Agro foil	94.69	5.31	3.53	0.27	4.79	1.81	0.28	72.70
CF	95.81	4.19	3.53	0.32	5.31	1.98	0.34	111.4
Year	2023							
CV	96.27	3.73	2.46	0.25	6.83	1.57	0.35	146.35
Jute	94.48	5.52	2.33	0.23	6.43	1.35	0.33	97.18
Hemp	96.06	3.94	2.92	0.23	6.28	0.82	0.31	65.80
PLA	95.80	4.21	3.28	0.10	6.61	1.34	0.33	115.73
Agro foil	95.35	4.65	2.52	0.20	6.49	0.88	0.32	76.28
CF	95.88	4.12	2.23	0.26	6.38	1.28	0.31	95.25

Where CF is the control field, CV is lettuce grown on soil mulched with viscose mulch, PLA is lettuce grown on soil mulched with polylactide mulch, N—nitrogen. P—phosphorus. K—potassium. Ca—calcium. Mg—magnesium. Fe—iron. d.m.—dry matter. Meaning ratio calculated from dry matter.

**Table 9:** Soil quality analysis for the years 2022 and 2023.

	pH		Humus, %	Organic C, %	Total N, %	P_2_O_5_, mg/100 g	K_2_O, mg/100 g
	H_2_O	1 MKCl					
Year	2022						
Before experiment	7.65	6.91	3.84	2.23	0.13	3.03	31.32
CV	7.58	7.00	4.68	2.71	0.60	9.15	27.00
Jute	7.78	7.02	3.55	2.06	0.28	9.39	19.39
Hemp	7.61	6.98	5.02	2.91	0.27	7.45	31.67
PLA	7.68	7.03	4.62	2.68	0.56	5.71	30.00
Agro foil	7.66	7.03	3.84	2.23	0.54	5.47	20.67
CF	7.54	6.95	4.39	2.55	0.58	10.55	29.67
Year	2023						
Before experiment	7.75	7.02	3.77	2.21	0.11	3.12	31.28
CV	7.77	7.10	3.95	2.68	0.28	3.86	27.10
Jute	7.90	7.15	4.32	2.75	0.28	18.16	41.29
Hemp	7.89	7.10	4.07	2.23	0.27	4.28	28.71
PLA	7.85	7.08	4.18	2.55	0.25	3.56	26.45
Agro foil	7.74	7.02	4.46	2.06	0.30	4.60	26.77
CF	7.85	7.08	4.30	2.91	0.29	5.40	28.07

Where CF is the control field, CV is soil mulched with viscose mulch, PLA is soil mulched with polylactid mulch.

**Table 10:** Total organic carbon (TOC) and total number of microorganisms (bacteria and fungi) in the soil after the lettuce harvest in the years 2022 and 2023.

Year	2022			2023		
	TOC, %	The Number of Colonies (CFU/g)		TOC, %	The Number of Colonies (CFU/g)	
		Bacterial	Fungi		Bacterial	Fungi
CV	3.84	1.15 × 10^7^	3.73 × 10^4^	2.76	6.90 × 10^6^	6.23 × 10^4^
Jute	3.61	7.77 × 10^6^	2.97 × 10^4^	3.11	1.02 × 10^7^	7.87 × 10^4^
Hemp	3.29	4.07 × 10^6^	6.17 × 10^4^	2.83	1.04 × 10^7^	6.73 × 10^4^
PLA	3.40	6.40 × 10^6^	3.10 × 10^4^	2.86	1.21 × 10^7^	2.94 × 10^5^
Agro foil	3.47	6.43 × 10^6^	2.77 × 10^4^	3.00	7.17 × 10^6^	5.90 × 10^4^
CF	3.42	8.90 × 10^6^	1.43 × 10^4^	2.86	4.40 × 10^6^	5.40 × 10^4^

Where CF is the control field, CV is soil mulched with viscose mulch, PLA is soil mulched with polylactide mulch.

**Table 11:** Weediness in the years 2022 and 2023.

Year	2022		2023	
	Mass of Weed, g	Weediness (%) Regarding to Control Field	Mass of Weed, g	Weediness (%) Regarding To Control Field
CV	0	0	0.84	0.07
Jute	3.49	0.51	43.28	3.80
Hemp	10.78	1.59	64.76	5.69
PLA	0	0	3.67	0.32
Agro foil	0	0	0.69	0.06
CF	678.95	100	1137.66	100

Where CF is the control field, CV is soil mulched with viscose mulch, PLA is soil mulched with polylactide mulch.

## Data Availability

Data are contained within the article.
